# Coping with Viral Diversity in HIV Vaccine Design: A Response to Nickle et al

**DOI:** 10.1371/journal.pcbi.0040015

**Published:** 2008-01-25

**Authors:** Will Fischer, H. X Liao, Barton F Haynes, Norman L Letvin, Bette Korber

Nickle et al. [1] recently reported a computational method for HIV-1 vaccine antigen design; here we compare this method with our previously published vaccine antigen design method [2], using several criteria that we believe to be important for a successful vaccine candidate [2]. The intent of both approaches is to design a set of vaccine antigens that could protect against diverse circulating strains of HIV-1 by eliciting broad T cell responses. T cells recognize short peptides of 8–12 amino acids, called epitopes, that are presented on the surface of infected cells by human HLA proteins. HIV-1 is highly variable, and both vaccine methods [1,2] attempt to provide coverage of most common variants of epitope-length fragments in HIV-1 proteins [3].

The two methods use very different computational strategies, but both achieve high coverage of potential epitopes. The COT+ method generates an antigen set consisting of one full-length synthetic sequence supplemented by a set of sequence fragments [1]. Initially, the Center of Tree (COT) sequence is computed based on a maximum likelihood phylogenetic tree inferred for a set of test sequences [4]. The “center” of the tree is identified as the point that minimizes the distances to the terminal branches, and the evolutionary model and tree topology are used to reconstruct the most likely sequence at that center point of the tree [4]. Like an inferred most recent common ancestral sequence [5], a COT sequence is an estimate of an historical entity, based on a concatenation of the most likely nucleotide at each position. The COT+ antigen design method uses the COT sequence as a foundation, and builds on it by sequential addition of protein fragments selected to provide enhanced potential epitope coverage [6]. These fragments are selected using a sliding window across all sequences; peptides are scored by the level of additional coverage they provide, and the highest-scoring fragments are added to the set, with fragments being overlapped where possible. The result is the COT+ sequence set: the full-length COT sequence plus a set of additional sequence fragments of varying lengths (Table 1, Figure 1). The summed length of all fragments are considered in units of “protein lengths”; Nickle et al. [1] illustrate the use of two protein lengths of peptides in addition to the COT protein, for a total of three gene length equivalents. They also provide an option of assembling the fragments into linear proteins (but this provided reduced population coverage relative to the basic COT+ method) [1].

**Table 1:**
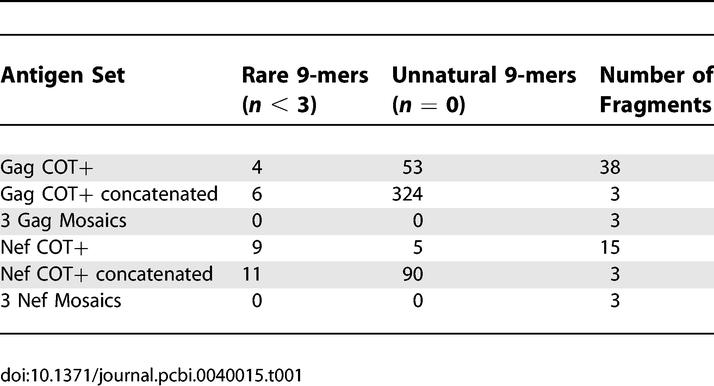
Number of Proteins or Protein Fragments Included in the Antigen Design, and the Number of Rare or Unique 9-mers in Various Protein Sets

**Figure 1:**
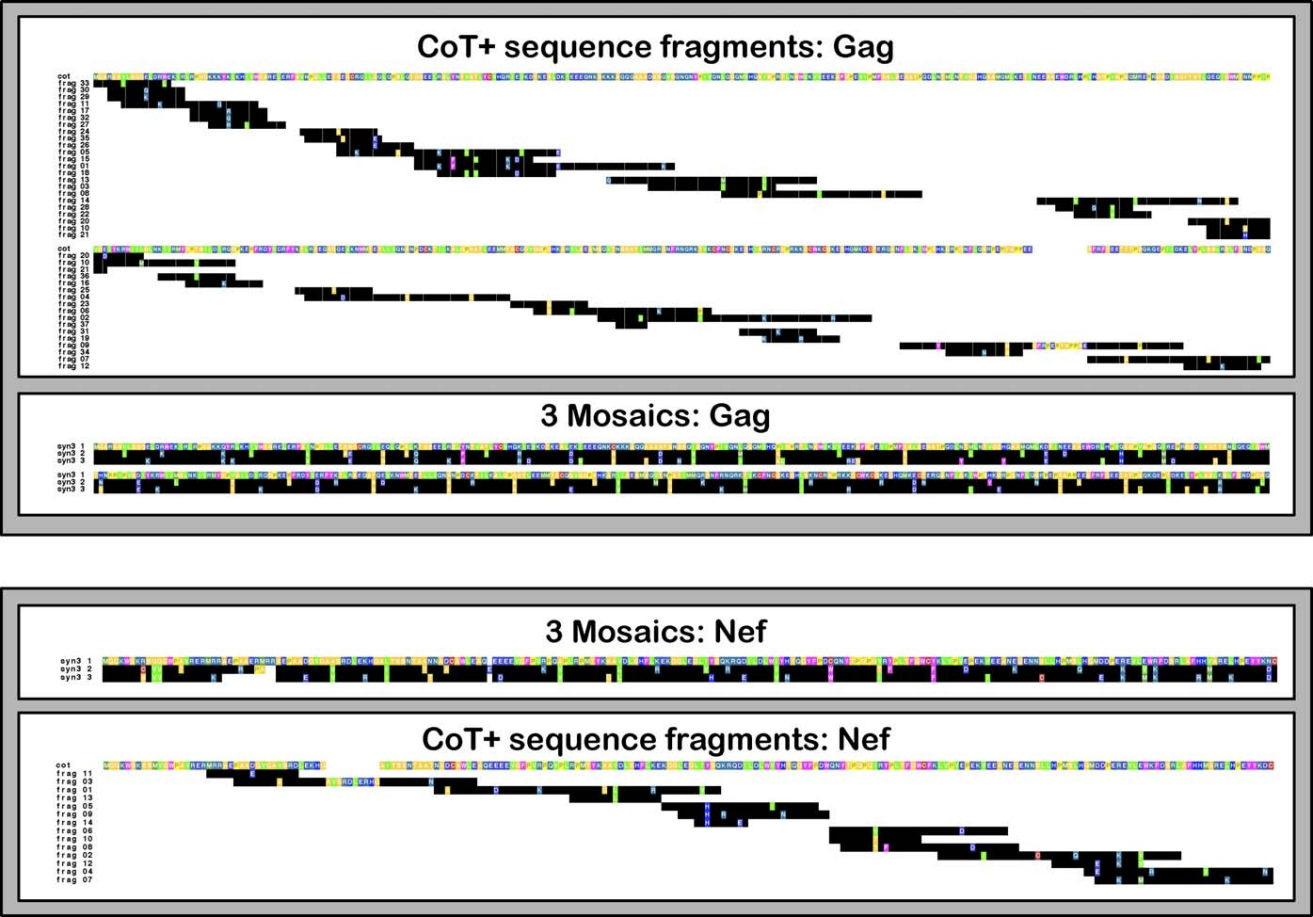
Relative Locations and Sequence Identities of Potential Vaccine Antigens The COT+ candidate vaccine sets represent one full-length COT protein plus numerous discrete sequence fragments located in various positions relative to the intact Gag protein; mosaics are full-length “native-like” proteins. For the COT+ sequence sets, the sequence on the top line is the COT sequence; additional peptide fragments are numbered by addition order, and plotted by location from N-terminus to C-terminus. Gag sequences are presented in two parts. For the mosaic sets, the sequence in the top line was arbitrarily chosen from the set, as the mosaics are designed as a combination of strains. For all sequence sets, amino acid residues identical to those in the top line sequence are shown with a black background; differences from the first sequence are shown in color, with different colors representing different amino acid classes; white space is used to represent gaps. The COT+ antigen sequences [1] were generously provided by J. I. Mullins.

In contrast, the mosaic method that we recently described [2] assembles a specified number of full-length protein sequences that in combination optimize coverage of epitope length peptides in a population. This method creates intact HIV proteins that have the potential for natural expression and processing of epitopes. We employed a genetic algorithm to generate sets of sequences by in silico recombination of natural protein sequences. Starting with randomly recombined natural sequences, the algorithm proceeds by a series of iterations of recombination and selection, optimizing for 9-mer coverage of the input sequences. The result is a small set of full-length protein sequences that collectively approach the upper bound on coverage attainable for a given number of proteins [2].

We designed a three-mosaic protein set using the same input data as Nickle et al*.* to directly compare the two methods using the same metric as Nickle et al. The mosaic set, despite being constrained to use full-length proteins, performed slightly better in terms of 9-mer coverage than three protein lengths using COT+ (Figure 2). The mosaics used three full-length protein sequences, however, compared to 38 fragments for Gag and 15 for Nef included in the optimal coverage COT+ antigen design (Figure 1, Table 1).

**Figure 2:**
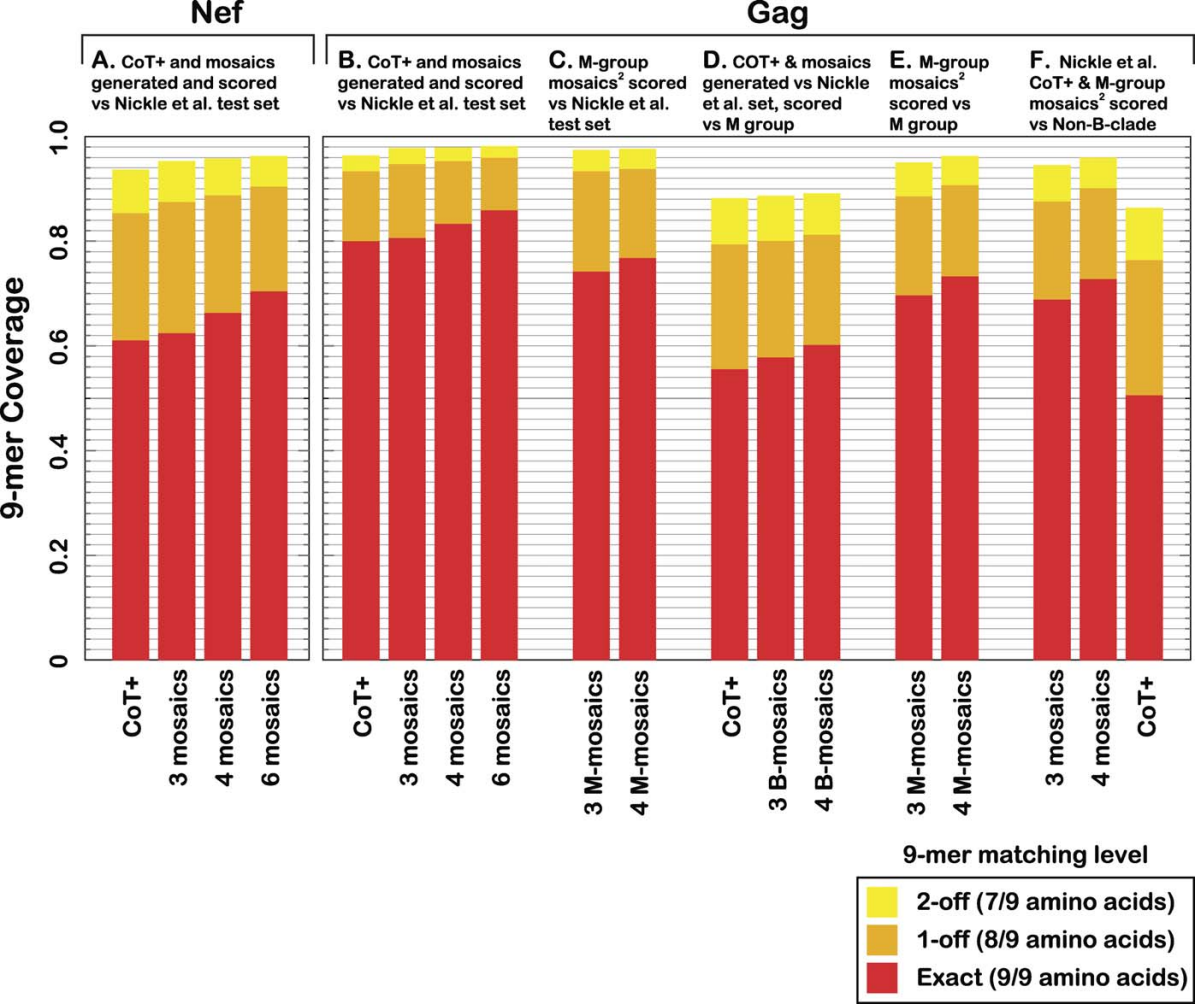
Comparisons of 9-mer Coverage for the COT+ and Mosaic Antigen Designs To allow direct methodological comparison, mosaic antigen sets were generated as in Fischer et al. [2], but based on the test set of 169 sequences used in Nickle et al*.* [1] for Nef (A) and Gag (B,D). Potential epitope coverage provided by the combination of proteins is indicated by the percentage of perfectly matched 9-mers in the protein alignment (red), the addition of those that match in 8/9 amino acids (orange), and the further addition of those that match in 7/9 amino acids (yellow). Three full-length mosaic proteins provide slightly better coverage than three gene lengths of COT+ protein fragments. For this class of methods, coverage may generally be improved by increasing the number of antigens, but with diminishing returns and at the cost of increased vaccine complexity and expense; the best strategy must realistically balance these factors. Previously published mosaic sequences (based on 551 Los Alamos sequences spanning the entire M group [2]) were scored against the 169-sequence Gag set (C), and the M group (E). To directly compare the coverage of each antigen set to subtypes other than B, the COT+ antigen set and the M-group mosaics were also scored against an M-group dataset from which all B-clade sequences had been removed (F). The M-group mosaic coverage of non-B clade proteins is 4%–5% less than the M-group coverage of B clade (C,F). Predictably, interclade coverage of the B-clade–optimized antigens drops dramatically (81% → 51% for COT+, F; B-clade mosaics slightly less reduced ([2], unpublished data). For the M-group global mosaics, in contrast, coverage is roughly equal for all clades; coverage of B-clade sequences by M-group mosaics is only 6%–7% below that of specific B-clade–optimized mosaics (B,C), and coverage of non-B sequences remains relatively high (F). All computer code for creating mosaics and assessing coverage, our previously designed mosaic antigens, and the datasets used were made publicly accessible upon the original publication [2].

The processing of antigens that ultimately results in the presentation of epitopes is not completely understood. Therefore, a critical issue for the COT+ method is a strategy for assembling the peptide fragments into antigens that would be practical for vaccine delivery. It is possible to simply concatenate the fragments, but this creates large numbers of spurious 9-mers at the junctions (up to eight at each junction; see Table 1), and may impact processing of embedded epitopes in the context of the assembled fragments.

Some recent experimental results illustrate that problems can occur at unnatural boundaries in polyproteins. We initially designed mosaic protein sets for Env, Gag, Pol, and Nef [2], although we highlighted creating mosaics from Gag and the conserved center of Nef [2]. Nef is highly variable, but is relatively conserved near the center of the protein, where the most intense and frequent T cell responses have been observed in the setting of natural infection [7]. Therefore, we excluded the variable regions of Nef and generated a single fusion protein comprising a mosaic full-length Gag plus the relatively conserved center of Nef. The Gag+central Nef fusion gene construct was cloned into a DNA vaccine vector and transfected into 293T cells. Protein expression was evaluated by Western blot analysis using HIV-specific MAbs (241-D, Gag; 6.2 and EH1, Nef). The mosaic polyprotein was larger than a Gag protein, as expected, and Gag-specific cellular immune responses were detected by IFN-γ Elispot assay in splenocytes from mice immunized with this polyprotein construct; however, Nef-specific responses were not detected. Furthermore, using recombinant Gag and Nef protein as coating antigens in an ELISA, we detected anti-Gag but not anti-Nef antibody responses in these mice. Thus, this Gag/Nef fusion construct expresses Gag and elicits Gag-specific immune responses, but does not induce anti-Nef antibody or T cell responses in vivo*.* These results demonstrate that unnatural linking of polypeptides can have unexpected and undesirable consequences, and that appropriate assembly of peptides can be a nontrivial endeavor.

Another important aspect of mosaic protein design is the explicit exclusion of unnatural or very rare epitope-length fragments [2]. While the excellent coverage of Mosaics illustrates that this approach captures more rare variants than other approaches (such as combining optimized natural strains or sets of consensus sequences) [1,2], unique or extremely rare variants are excluded at a user-specified rarity threshold. Including such fragments in a vaccine has the potential to elicit T cell responses that are unlikely to provide cross-protection against circulating strains, and could even divert vaccine-induced T cell responses from conserved epitopes that are relevant for protection. We therefore set a threshold such that all nine amino acid–length fragments that are incorporated into mosaic proteins are found a minimum of three times in the input data. In contrast, despite algorithmic “smoothing” [1], the COT+ protein fragments contain multiple rare and unnatural 9-mers (Table 1).

An exciting potential of this type of strategy is that it may provide an approach for making a global HIV vaccine. We previously compared mosaic vaccine design at three levels: regional, within-subtype, and global [2]; in contrast, COT+ was initially applied only to the B subtype [1]. The C subtype regional comparison was based on a large South African sequence dataset [8] compared to the non–South African C subtype Los Alamos database sequences; there was no discernable advantage in creating a South African–specific mosaic versus a global C subtype mosaic vaccine in terms of coverage of South African diversity [2]. We then compared B and C subtype mosaics with mosaics designed for the entire HIV-1 M group. We found that optimizing using one HIV subtype results in a dramatic reduction in coverage of other subtypes (generally about 25% for Gag and the Nef mosaics [2], also see Figure 2E for a COT comparison). In contrast, mosaics designed using a large and representative set of M group proteins from the Los Alamos database gave only slightly lower coverage for any given subtype compared to within-subtype optimized mosaics [2]—the tradeoff for this relatively small loss of coverage is the potential for much broader (i.e.*,* global) coverage. In Figure 2C, we show the 9-mer coverage of the M group Gag mosaics designed using the global set of M group sequences from the Los Alamos database. Coverage is comparable (within a few percent) for all subtypes tested [2] as well as for the test set from Nickle et al. [1] (Figure 2D).

In conclusion, although the COT+ method is an interesting and algorithmically creative suggestion for vaccine design, the Mosaic approach has several advantages. Nickle et al. [1] note that “more computational intensive approaches such as genetic algorithm searches . . . could also be brought to bear on the problem of antigen design.” Indeed, we have already applied such an approach and were able to achieve levels of coverage approaching the achievable upper bound, without sacrificing the linear protein sequence, and with the advantage of excluding rare epitope-length variants [2]. In contrast, the antigen sets generated by the COT+ method are either fragmented pieces of protein, or a subset of those fragments joined in a linear protein sequence that provides suboptimal coverage [1]. The most important result of our mosaic vaccine study is that it may provide a tractable approach for global vaccine design: high coverage of viral variability is presented in a feasible number of intact antigens for a vaccine cocktail. 

## References

[pcbi-0040015-b001] Nickle DC, Rolland M, Jensen MA, Pond SL, Deng W (2007). Coping with viral diversity in HIV vaccine design. PLoS Comput Biol.

[pcbi-0040015-b002] Fischer W, Perkins S, Theiler J, Bhattacharya T, Yusim K (2007). Polyvalent vaccines for optimal coverage of potential T-cell epitopes in global HIV-1 variants. Nat Med.

[pcbi-0040015-b003] Li F, Malhotra U, Gilbert PB, Hawkins NR, Duerr AC (2006). Peptide selection for human immunodeficiency virus type 1 CTL-based vaccine evaluation. Vaccine.

[pcbi-0040015-b004] Nickle DC, Jensen MA, Gottlieb GS, Shriner D, Learn GH (2003). Consensus and ancestral state HIV vaccines. Letter in response to Gaschen et al. [5]. Science.

[pcbi-0040015-b005] Gaschen B, Taylor J, Yusim K, Foley B, Gao F (2002). Diversity considerations in HIV-1 vaccine selection. Science.

[pcbi-0040015-b006] Jojic N, Jojic V, Frey B, Meek C, Heckerman D, Weiss Y, Schölkopf B, Platt J (2005). Using “epitomes” to model genetic diversity: Rational design of HIV vaccine cocktails.

[pcbi-0040015-b007] Frahm N, Korber BT, Adams CM, Szinger JJ, Draenert R (2004). Consistent cytotoxic-T-lymphocyte targeting of immunodominant regions in human immunodeficiency virus across multiple ethnicities. J Virol.

[pcbi-0040015-b008] Kiepiela P, Leslie AJ, Honeyborne I, Ramduth D, Thobakgale C (2004). Dominant influence of HLA-B in mediating the potential co-evolution of HIV and HLA. Nature.

